# An Update on the Role of Dietary Phytochemicals in Human Skin Cancer: New Insights into Molecular Mechanisms

**DOI:** 10.3390/antiox9100916

**Published:** 2020-09-25

**Authors:** Salman Ul Islam, Muhammad Bilal Ahmed, Haseeb Ahsan, Mazharul Islam, Adeeb Shehzad, Jong Kyung Sonn, Young Sup Lee

**Affiliations:** 1 School of Life Sciences, College of Natural Sciences, Kyungpook National University, Daegu 41566, Korea; dr_ssulman@yahoo.com (S.U.I.); muhammad786@knu.ac.kr (M.B.A.); amiable2012@hotmail.com (H.A.); sonnjk@knu.ac.kr (J.K.S.); 2 Department of Pharmacy, Faculty of Life and Environmental Sciences, University of Peshawar, Peshawar 25120, Pakistan; 3 Department of Chemical Engineering, College of Engineering, Dhofar University, Salalah 2509, Oman; mazharics@yahoo.com; 4 Department of Clinical Pharmacy, Institute for Research and Medical Consultations (IRMC), Imam Abdulrahman Bin Faisal University, Dammam 31441, Saudi Arabia; adeeb.shehzad@gmail.com

**Keywords:** skin carcinogenesis, melanoma, UV radiation, dietary phytochemicals, chemoprevention, free radicals

## Abstract

Human skin is continuously subjected to environmental stresses, as well as extrinsic and intrinsic noxious agents. Although skin adopts various molecular mechanisms to maintain homeostasis, excessive and repeated stresses can overwhelm these systems, leading to serious cutaneous damage, including both melanoma and non-melanoma skin cancers. Phytochemicals present in the diet possess the desirable effects of protecting the skin from damaging free radicals as well as other benefits. Dietary phytochemicals appear to be effective in preventing skin cancer and are inexpensive, widely available, and well tolerated. Multiple in vitro and in vivo studies have demonstrated the significant anti-inflammatory, antioxidant, and anti-angiogenic characteristics of dietary phytochemicals against skin malignancy. Moreover, dietary phytochemicals affect multiple important cellular processes including cell cycle, angiogenesis, and metastasis to control skin cancer progression. Herein, we discuss the advantages of key dietary phytochemicals in whole fruits and vegetables, their bioavailability, and underlying molecular mechanisms for preventing skin cancer. Current challenges and future prospects for research are also reviewed. To date, most of the chemoprevention investigations have been conducted preclinically, and additional clinical trials are required to conform and validate the preclinical results in humans.

## 1. Introduction

### 1.1. Structure of Skin

**Figure 1:**
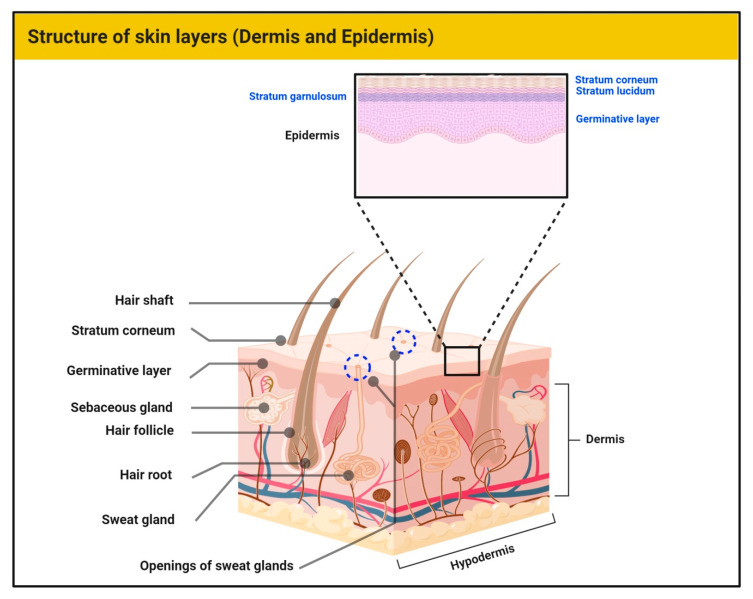
The human skin showing the layers of the epidermis and main structures of the dermis.

Skin represents the largest barrier of the human body to external insults. Histologically, skin is divided into three distinct layers, namely, epidermis, dermis, and hypodermis [1] (Figure 1). In general, skin provides protection from external pressures, serves as a source of percutaneous absorption, and has a significant role in thermoregulation. It is also responsible for the synthesis of macromolecules and aesthetics. The hypodermis consists of subcutaneous fatty tissue along with connective tissue. The dermis exhibits vascularity, neuronal ending, receptors (touch, nociception, and proprioception), hair, and glands. The extracellular matrix of the dermis contains connective tissue rich in collagen and elastin. The epidermis is the outer skin layer which is in direct contact with the atmosphere [1,2]. Approximately 80% of epidermal cells are keratinocytes. Due to its contact with the external environment, these cells undergo continuous wear and tear with constant replacement by newer keratinocytes from the basal epidermal layer. The separation between the epidermis and dermis is through the basement membrane [3]. Other specialized cells in the epidermis include melanocytes (melanin producing), Merkel cells (sensory), and Langerhans cells (immune role). All the cells of the skin perform complex functions, working to achieve and maintain constant homeostasis against various internal and external stimuli. In the case of damage to the skin cells, connective tissue, and other macromolecules, normal processes are dysregulated and give rise to various diseases, including mycoses, inflammatory diseases, and neoplasms [1,4,5].

### 1.2. Skin Carcinogenesis

**Figure 2:**
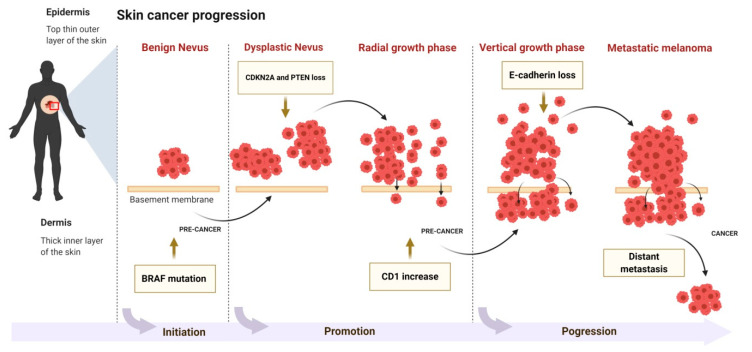
The molecular mechanism of skin cancer progression.

Neoplastic development and progression in the skin is a process consisting of three phases: initiation, followed by promotion and progression of tumor cells (Figure 2). The etiology of initiation may be either exogenous (chemicals, virus, ultraviolet (UV) radiation) or endogenous factors (inflammation). Damage caused by these factors to skin cells is irreparable when cells that cannot regain normal function advance to neoplastic progression [6]. Most ultraviolet B (UVB) radiation induces DNA damage that occurs as a transition from C to T or CC to TT in various genes including tumor suppressor gene p53 [7]. Ultraviolet A (UVA) radiation causes mutations via 8-oxodG. These are present as transitions from AT to CG in various genes that regulate cell cycle, apoptosis, and genomic stability (p53) [7,8]. Genetic mutation and continuous exposure to UV radiation leads to the advancement of initiated cells into the promotion stage [9]. Cells in the initiation stage replicate more vigorously and have less of a tendency to undergo apoptosis [9]. Actinic keratosis (AK) is an example of a precancerous lesion in humans that exhibits extensive mutations when compared with normal human skin cells [10]. 

Basal cell carcinoma (BCC), as well as squamous cell carcinoma (SCC), constitute the majority of non-melanoma skin cancers (NMSCs), with Caucasians being affected the most. Both arise from the cancerous transformation of normal keratinocytes [4,8]. Rogers et al. have estimated that approximately two million procedures in about 1.3 million individuals were carried out in 2012, and has increased in recent years [11]. Individuals usually develop invasive NMSCs in later stages of life due to its long latent period, even if the tumor-initiating mutations occurred during childhood. The incidence is less common at a younger age [11,12] and it is more frequent in men [13]. As the fifth most expensive cancer, NMSCs account for the expenditure of billions of dollars in health care and treatment [14]. Usually, patients diagnosed with NMSC die from secondary tumors in the lungs, prostate, or colon resulting from distant metastasis [15]. Therefore, early diagnosis and prompt treatment is highly recommended.

In the United States, most of the reported cases of BCC account for >3 million patients annually [11,12]. BCCs exhibit a slower growth rate with a rare chance of metastasis [16], though it does have a tendency to invade surrounding tissues [17]. In the US, SCC remains the second most prevalent skin cancer, accounting for 0.2–0.4 million cases each year. SCC has more of a tendency to metastasize, exhibiting a rate of approximately 4%. The annual number of deaths from metastatic disease is approximately 3000 [15,18,19]. The mortality rate increases in certain cases such as that of immunocompromised individuals [20]. Several studies have explored the transformation mechanism from normal keratinocytes to AK and finally, to SCC. It is believed that a greater period of latency occurs in SCC because AK is resistant to increases in harmful mutations that lead to SCC [10].

The current treatment for NMSCs includes complete eradication of the lesion while preserving the structural and functional architecture of the skin. Current treatment modalities involve surgical intervention including excision, curettage and electrodessication, cryosurgery, micrographic surgery, radiotherapy, and photodynamic therapy. Surgical procedures, such as the Mohs procedure, are preferred to other methods in cases of low-risk lesions [21]. However, the chance of recurrence is high with surgical procedures lacking a complete histological evaluation of the tumor to ensure its entire excision [22].

## 2. Dietary Phytochemicals for Skin Cancer Therapy

Dietary phytochemicals are present in plant-based food and exhibit certain nutritional and medicinal value, particularly in preventing various ailments and promotion of human health. Dietary phytochemicals are usually plant secondary metabolites and help proper functioning of human body. Studies have reported approximately 25,000 phytonutrients belonging to different classes of phytochemicals like polyphenols, phenolic acids, flavonoids, diarylalkanoids, carotenoids, lignans, anthocyanins, coumarins, terpenes, and sterols. In addition to various commonly-consumed vegetables and fruits, food items like nuts, beans, tea, and whole grains also contain significant amounts of phytonutrients. Owing to their inherent antioxidative characteristics, these phytonutrients contribute to enhance cardiovascular health, manage the diabetes, and offer cancer chemoprevention [23,24,25]. Although most dietary phytochemicals do not act like drugs for treating diseases, researchers suggests that their intake can be correlated to a number of positive health outcomes including reduced risk of cancer [24,26]. In this review we have covered various classes of dietary phytochemicals mainly in the context of their beneficial role against skin cancer. Our aim is to briefly introduce various aspects of dietary phytochemicals, highlighting their major sources, chemical classes, and major benefits in skin malignancy.

### 2.1. Resveratrol (RV)

RV is found in a variety of botanical sources such as blueberries, European pine, *Arachis hypogea* and *Reynoutria japonica* [27,28] (Table 1). Currently, RV is primarily acquired from red grapes. About 4 mg/L RV is found in grape juice [29]. Naturally, RV occurs in the trans- and cis- geometric stereoisomers. Although both isoforms exhibit similar activities, more studies have been conducted on its trans geometric isomer [30].

**Figure 3:**
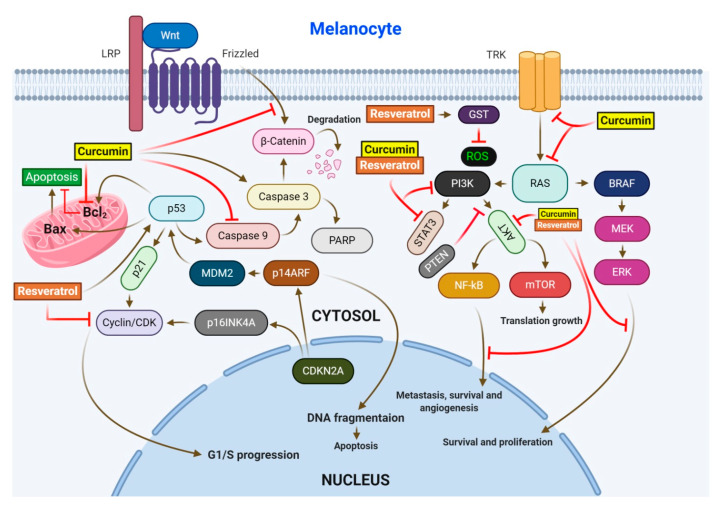
Regulation of the major cell signaling pathways by resveratrol and/or curcumin in melanoma. The four pathways regulated by these drugs include MAPK, PI3K/AKT, WNT/β-catenin, and the CDKN2/CDK4 tumor-suppressive pathway. Upon activation, these pathways lead to apoptosis, survival, proliferation, and angiogenesis.

RV has been reported to exhibit strong anticancer activity, acts as a ROS scavenger, and significantly reduces ROS levels in human skin fibroblasts [32,129]. It has been shown that along with other phytocompounds, RV suppressed tumorigenesis and decreased murine epidermal hyperplasia, while downregulating the expression levels of COX-2, Bcl-2, and p21 [130]. Kowalczyk et al. reported that during treatment of melanoma, RV reduced the viability of skin cancer cells and enhanced the cytotoxic effects of temozolomide [39]. It has also been demonstrated that RV inhibits the activity of redox factor-1, promoting the sensitivity of skin cancer cells to dacarbazine [131] (Figure 3).

Fuggetta et al. demonstrated that resveratrol exerts anti-melanoma effects both in vitro and in vivo. In this study, 7 μg/mL resveratrol prevented the proliferation of melanoma cells, inducing a 50% inhibition of cell growth [132]. Additionally, resveratrol inhibited the growth of A431 cells (SCC cells) [133]. Resveratrol induced cell death and downregulated cellular proliferation in FaDu, Cal27 and Det562 cells [134]. Kim et al. demonstrated that a 72-h treatment with 25 μM resveratrol decreased the synthesis of DNA [135]. An in vivo investigation revealed that 50 mg/kg body weight of oral resveratrol resulted in the inhibition of carcinoma cell growth, with over a 50% reductions in both tumor volume and murine weight [134]. Collectively, these findings suggest that resveratrol exhibits anticancer potential in vitro and in vivo.

Ndiaye et al. showed that because of fast clearance by the liver and intestine, RV exhibits poor bioavailability following oral administration [32]. Therefore, RV fails to stop tumorigenesis when administered orally to skin cancer-bearing mice [36]. Therefore, researchers have turned to topical or parenteral administration routes for RV. Moyano-Mendez et al. demonstrated that RV cream exhibits excellent properties including hydration, luminosity, and elasticity of skin [136]. Another study by Farris et al. demonstrated that RV improved healing of photodamaged skin after 12 weeks when combined with vitamin E and baicalin [136]. Further studies on the benefits and pharmacology of RV are ongoing and will hopefully yield promising results.

### 2.2. Curcumin

Botanical source of curcumin is the rhizome of *Curcuma longa*. It has been reported in multiple investigations that curcumin possesses remarkable anti-oxidative and anti-inflammatory properties, and exhibits significant therapeutic effects in atherosclerosis, ulcerative colitis, psoriasis, and Crohn’s disease [46]. Curcumin also exhibits anticancer activity by interacting and attenuating multiple protein targets [137]. A study by Dahmke and coworkers demonstrated the anticancer activity of curcumin against melanoma in mouse model. Curcumin upregulated miRNA-205-5p, a major player in the regulation of cellular proliferation [45] (Figure 3). In another study, curcumin was reported to induce cell death and inhibit proliferation and invasion by upregulating the expression of mmu-miR-205-5p and downregulating Bcl-2, PCNA, and JAK/STAT signaling [138]. Kim et al. demonstrated that curcumin reduces phosphorylation of Akt, S6K, ILGF-1 receptor, IRS-1, and 4EBP in mouse keratinocytes and significantly block chemical-induced skin cancer by DMBA in mice [139]. Zhao et al. reported that curcumin-induced arrest of cell cycle at G_2_/M transition checkpoint in A375 and C8161 cell lines. Curcumin was also found to induce autophagy in the melanoma cell lines. Moreover, curcumin blocked P70S6K activation and caused reduction in the expression of AKT and mTOR [140]. In a study by Wu et al., curcumin suppressed the STAT3 signaling pathway in A431 cells, leading to a significant reduction in cell invasion [141]. In WM-115 melanoma cells, curcumin stimulated the opening of mPTP, resulting in apoptosis [142]. Chinembiri et al. reported that curcumin inhibited the NF-κB pro-survival pathway, downregulated Bcl-2 expression, and activated the p53 tumor suppressor protein, resulting in cell death and inhibition of skin cancer [143]. 

Gupta et al. [44] studied the effect of curcumin against the SRB12-p9 skin cancer cell line in a mouse model. They demonstrated that oral curcumin at a dose 20 μM or higher effectively inhibited SCC growth and decreased the levels of pS6, a well-known downstream biomarker of the mTOR and MEK/ERK signaling pathways. The investigation further demonstrated that curcumin at a concentration of 20 μM or higher completely inhibited the proliferation of SRB12-p9 cells. The tolerability and safety of curcumin makes it a valuable phytomedicine for treating skin cancer.

### 2.3. Ursolic Acid

**Figure 4:**
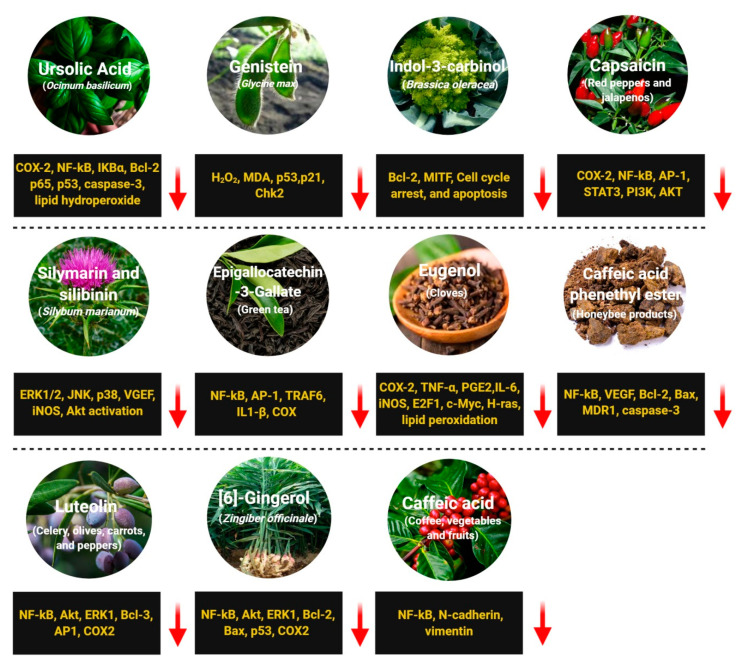
Regulation of various proteins, enzymes, and cellular signaling pathways by dietary phytochemicals in the prevention of skin cancer. Red down arrows indicate downregulation.

Ursolic acid (UA) is commonly found in basil, rosemary, thyme, apples, berries, oregano, peppermint, and prunes [144] (Figure 4). Studies have demonstrated that UA exhibits strong anti-inflammatory, antioxidant, chemopreventive, and anti-proliferative activity [145]. Several groups have shown that UA induces caspase-mediated apoptosis in melanoma cell lines [51,52]. Additionally, UA has been shown to modulate the G1 phase of the cell cycle by regulating p21WAF1 expression [49,146]. Checker et al. reported that UA suppresses the NF-κB signaling by inhibiting IκBα and p65 phosphorylation, which further decreases the expression of COX-2 enzymes, cyclin D1, and MMP-9 [147]. Manna et al. reported that, in addition to induction of apoptosis and cell cycle arrest, UA pre-treatment exhibited antioxidant activity in UVB-irradiated human lymphocytes [148]. Researchers are continuing to investigate the anticancer activity of UA, but to date, there have been no human clinical trials conducted in skin cancer. However, Both et al. administered UA liposomes to three healthy subjects and observed increased ceramide content in the skin [54]. Additional large-scale human skin cancer studies are needed to further reveal the molecular mechanism and efficacy of UA-based therapy.

### 2.4. Genistein

Genistein, 4′,5,7-trihydroxyisoflavone, is an isoflavone compound obtained from soybean [55]. Soybeans rich diets have existed for a long time and used for the treatment of cardiovascular disease, osteoporosis, and malignancies [56]. Studies have enumerated genistein as one of the most abundant phytoestrogens in soybeans and it exhibits potent anti-inflammatory, anti-oxidant, and anticancer effects [57,58]. The chemopreventive effects of genistein have been reported in various cancers including neuroblastoma, breast cancer, and non-melanoma and melanoma skin cancers [59]. Several studies have reported that genistein exerts anti-angiogenic effects, induces apoptosis, and decreases metastasis and tumor proliferation in various cancer cell lines [60,61]. In UV-induced sunburn in human, genistein prevented both UV-induced skin cancer and photoaging [62]. Wei et al. reported that genistein pretreatment followed by UVB-exposure in the epidermis of hairless mice prevents UVB-induced oxidative defect [62]. Moore et al. demonstrated the photoprotective characteristics of genistein in reconstituted human skin, in which genistein blocked the formation of UVB-induced pyrimidine dimer [149]. Additionally, in xenograft models, genistein (50 μM for four days) exhibited remarkable inhibitory effects against melanoma cells (40.2% loss of melanoma cells viability), while interfering with the cell cycle, blocking metastasis, and inhibiting tumor growth [63,150]. With respect to the underlying molecular mechanism of melanoma cell cycle progression inhibition by genistein, numerous studies have demonstrated that genistein targets p53, p21, and the Chk2 checkpoint kinase [64,65]. In addition to cell cycle regulation, genistein also promotes melanoma cell differentiation by stabilizing protein-linked DNA strand breakage [151,152]. It is necessary to conduct further clinical trials to optimize dosing, route of administration, and evaluate the efficacy of genistein for the prevention of skin cancer.

### 2.5. Indole-3-Carbinol (I3C)

I3C is a widely distributed member of the *Cruciferae* family that includes Brussels sprouts, broccoli, and cauliflower [153]. I3C has been reported to show activity against prostate, breast, and lung cancer [69,154,155,156] Sarkar et al. reported that IC3 (60 μmol/L) and its in vivo dimeric product 3,3′-diindolylmethane (30 μmol/L) showed anticancer potential in prostate cancer cell lines through the regulation of cell cycle, cell proliferation, cell death, and transcriptional activities. It was postulated that the downregulation of NF-κB, MAPK, Akt, and Bcl-2 signaling were responsible for I3C-induced cell death in prostate cancer cells. Additionally, I3C also sensitized the prostate cancer cells to cisplatin (17 nmol/L) treatment [154]. In a similar study, Rahman et al. investigated the actions of I3C in Her-2/neu over-expressing MDA-MB-435 breast cancer cells. This investigations revealed I3C (30–100 µM for 24–72 h) affected the ratio and cellular localization of the Bcl-2 and Bcl-XL (anti-apoptotic proteins) and Bax (pro-apoptotic protein), producing conditions that favour breast cancer cells death [155]. During an investigation regarding the effect of I3C on human lung carcinoma A549 cells, Hee-Sook Choi et al. found that I3C (100–500 µM for 48 h) effectively decreased cells proliferation, elevated the formation of apoptotic bodies, and arrested the cell cycle at G_0_/G_1_ phase. Moreover, I3C also enhanced the protein levels of cyclin D1, p21, and p-p53, and upregulated FAs at mRNA levels. The study suggested I3C as a potent therapeutic agent against lung cancer [156]. I3C induced apoptosis and arrest of cell cycle in UVB-sensitized human melanoma cells by downregulating Bcl-2 and MITF [70,71]. Moreover, I3C also inhibited human melanoma cell proliferation by regulating PTE degradation [72]. Using a mouse model, Christensen and LeBlanc showed that I3C (333 of 500 mg/kg/day) increased chemotherapeutic drug sensitivity [157]. Clinical studies will be required to further prove the safety and efficacy of I3C in skin cancer patients.

### 2.6. Capsaicin

Capsaicin, *trans*-8-methyl-*N*-vanillyl-6-nonenamide, is the most popular spice in the world. Capsaicin is a phenolic acid, acting as the key pungent constituent in red chili peppers and provides spiciness to red peppers and jalapenos. There is conflicting evidence as to whether capsaicin is a carcinogen or a chemopreventive agent [73]. Hwang et al. observed a pro-carcinogenic effect of topical capsaicin as it promoted skin carcinogenesis through the activation of EGFR and COX-2 in mice treated with 12-O-tetradecanoylphorbol-13-acetate (TPA) [74]. In contrast, Wang et al. reported that topical capsaicin did not promote the growth of murine skin tumors, and also slightly blocked the formation of papilloma in mice [158]. An investigation correlated the chemopreventive effect of capsaicin to the stimulation of cell death, cell cycle arrest, and decrease of cell proliferation via the inhibition of COX-2, NF-κB, AP-1, and STAT3 expression [159]. Moreover, capsaicin has been reported to induce cell death in human cutaneous SCC cells by inhibiting mitochondrial activity [75]. Shin et al. described the potent anti-migratory activity of capsaicin against highly metastatic melanoma cells by the inhibition of Akt and PI3-K signaling [76]. Marques et al. reported that capsaicin synergistically induced apoptosis with HA14-1 in melanoma cells [77]. These findings warrant further intensive studies to clarify the capsaicin role in skin cancer.

To date, there have been no studies describing the topical use of capsaicin in skin cancer. Therefore, we have focused our discussion on the topical use of capsaicin for other ailments. Topical capsaicin has been investigated in multiple double-blind placebo-controlled studies for treating chronic neuropathic or musculoskeletal pain. It was shown that, compared to the placebo, one out of three patients utilizing capsaicin experienced high degree of adverse events such as stinging, erythema, or burning [78]. Therefore, a notable limitation would be for the use of topical capsaicin against skin cancer. Different formulations of capsaicin, with or without other ingredients, may decrease the incidence of local adverse events.

### 2.7. Silymarin and Silibinin

Silibinin, a potent phytochemical, is obtained from milk thistle and is considered the major bioactive molecule in the silymarin complex. Because of poor bioavailability, the application of silibinin has been restricted. To improve absorption, researchers have attempted to develop new formulations in the form of nanosuspensions [80]. Silymarin has been used for the treatment of liver diseases [160]. Additionally, multiple clinical trials have demonstrated the chemotherapeutic potential of silymarin on a variety of cancers including skin malignancy [81]. It has been reported that silymarin exerts chemotherapeutic activity by inhibiting TPA-induced tumors in murine skin. Moreover, silibinin was demonstrated to show strong anticancer activity by targeting the CDK pathway and subsequently arresting the cell cycle [82]. During this investigation, researchers found that silibinin inhibited angiogenesis by targeting VEGF receptors and iNOS [83,89]. Silibinin has also been shown to trigger caspase-mediated apoptosis through the extrinsic and intrinsic pathways [85,86].

It has been shown that silymarin prevented UV radiation-induced skin cancer in a mouse model of photo-carcinogenesis [87,161]. Moreover, silymarin was found to block UVB-induced sunburn, reduce catalase activity, and stimulate the expression of COX and ornithine decarboxylase [88]. Studies have also shown the potential anticancer action of silibinin in targeting the MAPK-mediated signaling cascade [91,162]. Mallikarjuna et al. reported that both oral and topical silibinin blocked UV-induced MAPK, *p38*, JNK, and Akt activity in SKH-1 murine skin [90]. Additionally, it was demonstrated that silymarin remarkably blocked the β-catenin accumulation in human melanoma cells, which subsequently led to the inhibition of cell migration [93,163]. It can be concluded from these investigations that silymarin and silibinin are effective chemotherapeutic and chemopreventive agents against skin cancer, and additional clinical trials of silymarin with respect to its bioavailability and toxicity are needed.

### 2.8. Epigallocatechin-3-Gallate (EGCG)

EGCG is the major polyphenol compound from green tea. EGCG is the most famous anti-oxidant, anti-inflammatory, and anti-proliferative polyphenol among the green tea phenols [101]. An investigation reported that anti-inflammatory potential of EGCG were correlated with the suppression of COX and lipoxygenase activity, which lowered skin tumor burden and reduced hyperplasia and epidermal edema [101,164]. A study reported that, in human skin, topical EGCG blocked UV-induced nitric oxide and hydrogen peroxide in both the epidermis and dermis [101]. Such a reduction may be linked to the downregulation of MAPK signaling pathways [165]. Others have suggested that anti-proliferative mechanisms include the regulation of NF-κB, AP-1, angiogenesis, and cytotoxic T cells [99,100,166].

A study reported that EGCG sensitized skin cancer cells to interferon-induced growth inhibition, reduced the proliferation of cells, and induced apoptosis [98]. Furthermore, it was noted that concomitant treatment of EGCG with interferon showed strong effect than either agent alone. The possible underlying mechanisms demonstrated by this study include downregulation of inflammasome and NF-κB activity, which decreased interleukin-1β secretion and tumor growth, respectively [97]. Zhang et al. reported that EGCG inhibited the migration and invasion of melanoma cells by abolishing TRAF6 activity [96].

EGCG has undergone several small human trials for skin cancer chemoprevention. Currently, an important issue under consideration is oral versus topical administration of EGCG. It was shown in a study that green tea constituents administered to mice through oral or parenteral routes effectively inhibited UV-induced skin papilloma [167]. In contrast, an investigation demonstrated that tumor reducing outcomes in mice were obtained only by topical EGCG, whereas oral administration was ineffective [168]. It has been postulated that the discrepancy between the topical and oral administration of EGCG is due to inadequate EGCG supply to the skin after oral ingestion. An investigation confirmed the protective activities of topical green tea phenols against UV radiation-induced erythema in a cohort of human volunteers [169]. Furthermore, it was demonstrated in a single-blind, randomized clinical trial of 50 volunteers that, compared to the placebo group, supplementation of oral green tea extract with vitamin C did not exhibit effectively decreased leukocyte infiltration or skin erythema [170]. During a double-blind phase II randomized clinical trial, 51 volunteers with AK were administered topical EGCG for 12 weeks. The researchers did not find any significant difference between the EGCG and placebo groups at the end of the investigation. It was postulated from the trial that topical EGCG, possibly because of poor bioavailability, may not have been active in the formulation [152]. Collectively, it may be concluded that for skin cancer prevention, topical ECGG is more effective compared with an oral formulation. However, the optimal formulation for topical EGCG requires further investigation. Moreover, EGCG role, as a potential synergistic treatment for skin cancer, remains another important area for further study.

### 2.9. Eugenol

Eugenol is a phenolic compound which is abundantly found in cinnamon, basil, bay leaves, cloves, and nutmeg. Eugenol may be administered at different dosages through various routes. It exhibits antioxidant and anti-proliferative activities through different mechanisms. For example, as an antioxidant, eugenol inhibits ROS formation and lipid peroxidation [171]. Moreover, topical eugenol blocks the inflammatory response by regulating various proinflammatory molecules including COX-2, PGE_2_, iNOS, IL-6, TNF-α, and NK-κB [102]. Pal et al. demonstrated that topical and oral eugenol decreased the incidence of papilloma development in mice [104]. Another study showed that eugenol downregulates the c-Myc and H-ras genes, modulates the levels of p53, and stimulates cell death by decreasing E2F1 synthesis [103,104].

Esmaeili et al. formulated a 2% nanoemulsion of eugenol for topical use, which at 1.5 h, demonstrated superior anti-inflammatory activity compared with topical piroxicam [105]. Additional skin permeation investigations will be required to understand the anti-inflammatory, antioxidant, and anticancer activities of eugenol as well as for the development of more potent formulations.

### 2.10. Caffeic Acid Phenethyl Ester (CAPE)

CAPE is the major bioactive molecule of propolis, which is obtained from honeybee products. Multiple studies have reported the in vitro and in vivo anticancer potential of CAPE against various cancers including lung cancer, colon cancer, glioma, pancreatic cancer, breast cancer, hepatocellular carcinoma, gastric cancer, cholangiocarcinoma, and melanoma [107,108,109,172,173,174,175,176]. A study reported the anticancer, anti-inflammatory, and immunomodulatory effects of CAPE in vitro [177]. CAPE effectively blocked TPA-induced skin papilloma in mice. VEGF and MDR-1 levels were downregulated upon CAPE treatment. CAPE also regulated cell cycle and cell death via NF-κB [106]. Chen et al. reported that CAPE attenuated the expressions of Bcl-2, Bcl-2, and caspase-3 in leukemia cell lines, which subsequently led to cell death [178]. Yordanov et al. also showed that CAPE exhibits antioxidant effects on murine skin at lower doses [179]. In short, CAPE may act as a chemopreventive agent for skin cancer, however, clinical trials are required to verify the overall efficacy.

### 2.11. Luteolin

Luteolin is abundant in celery, olives, carrots, and peppers. Studies have demonstrated that luteolin exhibits anticancer activity through various molecular mechanisms including angiogenesis inhibition, caspase-mediated apoptosis, and by sensitizing cancer cells to anticancer drugs [114]. Luteolin has been found to stimulate melanogenesis and decrease the aggressiveness of skin cancer cells through regulation of the β3-integrin and focal adhesion kinase signaling cascade [113,180]. Studies have shown that luteolin induces cell death and prevents cancer cell growth by regulating Bax, Bcl-2, and ERK1/2 signaling [110,111]. These findings suggest the luteolin may be a potent anticancer molecule, however, additional investigations are needed to better understand the efficacy and pharmacology of luteolin.

### 2.12. [6]-Gingerol

[6]-Gingerol, a pungent phenol, is obtained from the roots of the *Zingiber officinale* ginger plant. Approximately two decades ago, Park et al. demonstrated that [6]-gingerol significantly inhibits skin papilloma formation [181]. Subsequent studies have shown that [6]-gingerol lowers the activity of epidermal ornithine decarboxylase, inhibits COX-2, and downregulates the activation of NF-κB by modulating MAPK activity [115,181]. Moreover, [6]-gingerol exhibits antioxidant action by decreasing UV-induced ROS generation, activation of Fas as well as caspase-3, -8, and -9 levels [116]. Other possible molecular mechanisms are the modulation of AP-1 DNA binding potential and regulation of survival factors such as Bcl-2, Bax, and p53 [117,118]. To date, there are no published human trials regarding the topical use of [6]-gingerol. Efforts have been made to incorporate [6]-gingerol into solid lipid nanoparticles with improved chemical stability for topical use, which may provide a feasible and stable option for evaluating efficacy [119].

### 2.13. Caffeic Acid (CA)

CA is widely distributed in coffee, fruits, and vegetables. CA is a polyphenolic bioactive compound exhibiting remarkable antioxidant, anti-inflammatory, and anticancer activities [120,121]. A study reported that, in keratinocyte cells, CA effectively inhibited colony and EGF-induced tumor formation [124]. Furthermore, it has been shown that CA reduced the cancer stem cells migration cancer stem cells by enhancing the phosphorylation of p38 and inhibiting NF-κB/snail signaling. It was further demonstrated that p38 blocked the binding potential of NF-κB to the promoter of the snail gene, resulting in inhibition of snail expression. Additionally, CA treatment prevented the epithelial-mesenchymal transition (EMT) in human keratinocyte tumors, which was evident from increased levels of E-cadherin, whereas *N*-cadherin and vimentin levels were downregulated. These studies reveal that CA plays a role in preventing the invasion and migration of cancer cells [105,124]. Researchers remain optimistic for the development of new formulations of CA for skin cancer treatment.

## 3. Role of Whole Fruits and Vegetables in Skin Cancer Prevention

**Figure 5:**
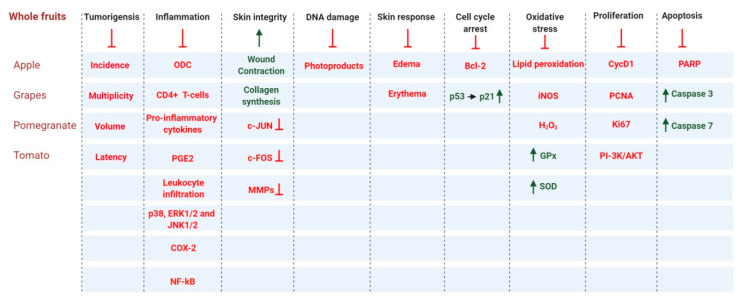
Whole fruit and vegetable products contribute to the prevention of skin carcinogenesis by regulating a broad range of cell signaling pathways. Arrows and dark green text indicate activation and/or upregulation, and red lines with a blunt end and red text indicate inhibition and/or downregulation.

Fruits and vegetables have high nutrient content. Whole fruits can be fresh, dried, canned, or frozen. Dieticians recommend that fruits should be ingested whole or in 100% pure juice form, which preserves their nutrient-dense nature [182]. Studies have shown that fruits contain various phytonutrients with anti-carcinogenic, anti-mutagenic, antioxidant, and anti-inflammatory properties [183,184,185,186]. Here, we discuss the benefits whole fruits and vegetables in the context of skin cancer. This section includes multiple in vitro and in vivo studies on the effects of whole fruit and fruit extracts against skin cancer (Figure 5).

### 3.1. Apple (Malus pumila)

Apples are one of the largest sources of fruit phenolics in the United States [187]. Apple skin contains more phenolic content compared with the remainder of the fruit [188]. The apple flesh contains caffeic acid, procyanidins, and catechins, whereas the peel contains all of these plus quercetin glycoside [188]. Ding et al. conducted a study to determine the chemopreventive effects of fresh apple peel extract against DMBA-TPA-induced skin tumorigenesis in a transgenic mouse model. Mice were given apple peel extract for two days in drinking before starting a dose of DMBA, and then continued till the end of the investigations [189]. The mice were sacrificed after 20 weeks of biweekly TPA application. The mice ingesting the apple peel extract exhibited a greater than 50% inhibition in the number of papillomas, accompanied by a decrease in tumor volume [189]. This study further revealed that the reduction of tumorigenesis was relative to a concomitant decrease of ROS and regulations of MAPK and AP-1 [189]. George and Rupasinghe tested the effect of a flavonoid-rich ethanolic extracts of the Northern Spy apple cultivar against carcinogen-induced toxicities in normal human bronchial epithelial cells. The apple flavonoids reduced total ROS generation, blocked carcinogen-induced oxidative DNA damage, and facilitated DNA repair mechanisms [190]. To date, very few studies, mostly in vitro, are available in this area and additional investigations in animal models and humans will be necessary (Table 2).

### 3.2. Pomegranate (Punica granatum)

Pomegranate fruit contains both types of true tannins: anthocyanins (condensed tannins) and hydrolyzable tannins [198,211]. Its effect on skin diseases including cancer have been studied by several research groups. The administration of pomegranate fruit extract (PFE) via a topical route (2 mg in acetone) to CD-1 mice, followed by TPA application at a dose of 3.2 nmol/mouse after 30 min, resulted in the inhibition of the TPA-induced inflammatory response [199]. A reduction in skin erythema, swelling, and COX-2 activity was also observed. Furthermore, a reduction in MAPK signaling was evident. PFE inhibited tumor growth initiated and promoted in CD-1 mice by DMBA and TPA, respectively. The latency period of the tumor was extended and approximately 20% of the PFE-treated animals remained tumor-free [198]. This study demonstrates the protective effects of PFE against inflammation, and tumor development and progression. Similarly, antineoplastic effects were observed by Hora et al. in mice that were administered 5% pomegranate seed oil [199]. Gil et al. induced skin damage in SKH-1 hairless mice by UVB radiation. In one dosing schedule, PFE was administered in drinking water for 14 days prior to a single dose of radiation, whereas in another dosing schedule, radiation exposure was given on alternate days for seven days. PFE caused a reduction in skin edema, leukocyte migration at the inflammatory site, cell proliferation, and COX-2 expression. Using the first dosing schedule, PFE-treated mice exhibited increased expression of p53 and p21 accompanied by enhanced repair of CPDs and 8-oxodG. A lower proliferative index was observed with both dosing schedules as determined by expression of the proliferating markers, PCNA [200,201] and cyclin D1 [201]. UVB radiation also increased the activation of NF-κB. The study with the second dosing schedule also evaluated the upstream proteins responsible for activating MAPK and NF-κB [200,201] and it was found that PFE reduced the expression of ERK1/2, JNK1/2, and p38. Therefore, PFE exerts its protective effect by attenuating multiple intracellular signalling pathways involved in various cellular processes including survival, proliferation, apoptosis, and the inflammatory response [201]. Pomegranate juice and oil have also been evaluated for their protective effects. Studies have revealed photoprotective activity against UVB-induced skin damage in NHEK [212], HaCaT keratinocytes [192], and human skin (reconstituted) [194]. PFE has also been found to inhibit the phosphorylation of c-Jun and c-Fos, thereby rendering them inactive. PFE inhibits MMP protein and c-Jun, c-Fos, and MMPs are involved in the breakdown of collagen in connective tissue [192,194]. Overall, these studies establish a protective role for pomegranate in inflammation and skin cancer.

### 3.3. Tomato (Solanum lycopersicum)

An abundance of carotenoids in tomato has been attributed to its skin protective effects. Lycopene, the major carotenoid found in tomatoes, was reported to function as a free radical scavenger [213]. Stahl and co-workers have suggested that lycopene, along with other carotenoids and noncarotenoids in tomatoes, are responsible for eliciting photoprotective effects against UV radiation [214]. Kopec et al. examined the photoprotective effects of tomato in a gender-based manner. SKH-1 hairless mice were exposed to UV-mediated skin damage followed by an evaluation of the effects of tomato. UVB exposure was applied in a single dose whereas tomatoes were provided for 10 weeks. The results indicated that the levels of carotenoids in skin and blood were higher in female mice. With respect to the photoprotective mechanism, UVB-induced carotenoids were associated with a reduction in inflammation and CPDs [202]. In a study by Cooperstone et al., mice were provided with a tomato-containing diet for 35 weeks. The mice were exposed to radiation three times a week at a dose of 2240 J/m^2^. In male mice, papilloma were produced at 6–10 weeks compared with 10–12 weeks for the female mice. Interestingly, it was observed that male mice treated with a tomato diet exhibited a lower number of tumors compared with the control group, whereas no differences were observed in the female cohort [205]. These results are encouraging, but further investigations are needed to analyze the photoprotective effects of tomato constituents in other in vitro and in vivo models.

### 3.4. Grape (Vitis vinifera)

Filip et al. used extracts from grape seeds to determine its protective effects against UVB radiation. The extracts (4 mg/cm^2^ per mouse) were topically applied 30 min before a single dose of 20 mJ/cm^2^ UVB radiation to female SKH-1 hairless mice. The extract was found to reduce lipid peroxidation, nitric oxide, and reduced the activity of caspase-3. Hence, the seeds were effective in reducing oxidative stress induced by UVB radiation [215]. In another study, grape seed extracts were analyzed for their photoprotection by increasing UVB exposure continuously for 10 days at a dose of 240 mJ/cm^2^. The extract was found to reduce CPDs, cell proliferation (hyperplasia), cytokine release (inflammation), and oxidative stress [216]. Jang et al. used grape stem extracts to determine the effects against UV-induced skin damage. C57BL mice were given UVB radiation doses three times a week for three weeks at dose of 120 mJ/cm^2^. Extracts were provided for one week before irradiation and continuously throughout the duration of the study. The observed effects included a reduction in lipid peroxidation, reduced neutrophil and mast cell migration to the site of exposure, and reduced COX-2 expression [217]. In a study by Cho et al., a reduction in oxidative stress was observed through increased activity of glutathione peroxidase and superoxide dismutase. In addition, the skin architecture was preserved and DNA damage was also prevented. The duration of UVB radiation was three times a week for one month in male Balb/c mice and the extracts from Muscat bailey A grape were applied topically. A reduction in the inflammatory response was also observed as evidenced by reduced leukocyte migration and reduced proinflammatory cytokine production [206]. In a study by Perde-Schrepler, immortalized HaCaT keratinocytes were treated with a Burgund mare variety of red grapes. A protective effect against UV radiation (25–300 mJ/cm^2^) was observed at concentrations of 10–20 μg/mL. An increase in skin viability and a reduction in apoptosis, lipid peroxidation, and DNA damage were observed [195].

Kobayashi et al. evaluated the effects of Y grape juice and its fractional extract in ethyl acetate on six-week-old SENCAR mice. Edema was induced using TPA, whereas carcinogenesis was induced with DMBA. Topical and oral treatment with the extracts reduced edema, whereas DMBA-TPA induced carcinogenesis was reduced to significant extent as evidenced by decreased tumor number, tumor incidence, and COX-2 action [218]. An investigations reported a different treatment protocol in a similar mouse model. The treatment plan included initial pre-treatment with resveratrol followed by the administration of freeze-dried grape powder (GP) or continued oral administration of GP in AIN-93G diet for a period of two weeks before chemical induction of tumors. After 12 weeks, both treatments reduced tumor number, COX-2 expression, and DNA damage [210]. Since 90% of skin cancers are linked to UV rays, chemically-induced tumors are less significant [209]. In another study of UV induced tumors in SKH-1 female mice, grape seed (rich in proanthocyanidins) was evaluated for its protective effects. The dose and duration of radiation was 180 mJ/cm^2^ three times a week for 24 weeks. A reduction in radiation-induced inflammatory mediators and COX-2 expression was observed [219]. Singh et al. subjected SKH-1 female mice to 180 mL/cm^2^ of UVB rays twice a week for 28 weeks. The diet included a 0, 3, or 5% concentration of freeze-dried GP. The diet resulted in increased apoptosis and reduced lipid peroxidation and proliferative markers [220]. The treatment also increased NER-facilitated repair of the skin by reducing the level of CPDs [220]. Overall, grapes and their components exert promising activity as naturally-occurring anti-inflammatory and anti-neoplastic agents.

## 4. Limitation, Safety Consideration, and Future Prospects of Dietary Phytochemicals

Dietary phytochemicals have experienced many challenges including low bioavailability. In our daily diet, we consume a significant amount of these phytochemicals. They are readily digested and eliminated by our body, resulting in a short-lived pharmacologic window [221]. Researchers are striving to develop new approaches that will increase the stability of dietary phytochemicals. Noteworthy strategies include the development of a stable dosage form of dietary phytochemicals, such as microparticles or nanoparticles, exhibiting increased stability and antioxidant properties [222]. Iqbal et al. demonstrated that EGCG showed increased stability and high absorption in the intestine when green tea extract was coated with chitosan [223]. Yadav et al. observed enhanced stability and antioxidant activity of bovine serum albumin-coated catechin and epicatechin nanoparticles [224].

The absence of target specificity is another important challenge for dietary phytochemicals in cancer treatment. It has been shown that phytochemicals exert pleiotropic effects at the cellular level, whereas cancer cells activate other cell signals resulting from the failure of targeted therapy [225]. Scientists strive to utilize alternative strategies for managing these obstacles, including novel formulations for targeted delivery of phytochemicals, formulating semi-synthetic derivatives and analogs of phytochemicals, and development of novel drug delivery systems to enhance the effectiveness, protective characteristics, bioavailability, and pharmacokinetics of phytochemicals in humans [28,226]. Further studies are needed to meet the challenges of topical skin cancer formulations which include skin penetration, optimum drug concentration, stability, dosing strategy, and sustained drug release following topical application.

## 5. Conclusions

Dietary phytochemicals have several advantages for skin cancer prevention because they are readily available, cost-effective, and well tolerated. The use of dietary phytochemicals have an inverse relationship with skin cancer [227,228]. They are natural antioxidants, elevating the levels of antioxidant enzymes, CDKs, cyclins, p53, p21, and Bax. Moreover, they scavenge ROS, and decrease various molecular targets including EGFR, Notch-1, ERK, MAPK, NF-kB, STAT, β-catenin, PI3K, AKt, and mTOR [153,161,228]. Dietary phytochemicals can also inhibit the proliferation of established skin cancer cells by arresting the cell cycle, preventing metastasis and angiogenesis, suppressing EMT, regulating epigenetic alterations, and downregulating MMPs and COX-2 enzymes. Further investigation, including short-term human studies, may be beneficial in assessing the human relevance of the preclinical data. Moreover, skin cancer chemoprevention investigations involving whole fruits and vegetables are required in the humans who are at high risk, such as individuals with compromised immunity. Preclinical studies in models of high-risk skin carcinogenesis may show beneficial effects. Furthermore, whole fruits and vegetables may also be combined with existing therapeutic strategies for the better management of skin cancer.

## Figures and Tables

**Table 1:** Dietary phytochemicals and their proposed molecular mechanisms in the prevention of skin cancer.

Dietary Phytochemical	Source	Molecular and Structural Formula	Actions/Targets	Reference
Resveratrol	Grapes, peanuts, Japanese knotweed, blueberry, Scots pine and *Reynoutria japonica*	C_14_H_12_O_3_ 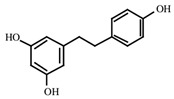	Anticancer; anti-inflammatory; anti angiogenic; Reactive oxygen species (Ros) scavenger; anti proliferative	[31,32,33,34,35,36,37,38,39,40,41,42,43]
Curcumin	Turmeric	C_21_H_20_O_6_ 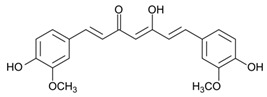	Anti-oxidant; anti-inflammatory; anticancer; anti proliferative; cell cycle regulator	[44,45,46,47,48]
Ursolic acid	Basil, rosemary, thyme, apples, berries, oregano, peppermint, prunes	C_30_H_48_O_3_ 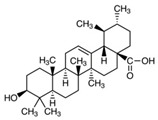	Anti-inflammatory; antioxidant; chemopreventive; anti-proliferative	[49,50,51,52,53,54]
Genistein	Soybean	C_15_H_10_O_5_ 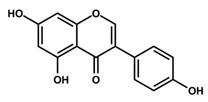	Cell cycle arrest; anti-inflammatory; anti-oxidant; anticancer	[55,56,57,58,59,60,61,62,63,64,65,66]
Indole-3-carbinol	Brussels, broccoli, cauliflower, sprouts	C_9_H_9_NO 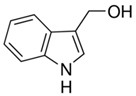	Anticancer	[67,68,69,70,71,72]
Capsaicin	Pepperoni, jalapeno, piri-piri, habanero peppers	C_18_H_27_NO_3_ 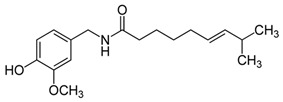	Anticancer; chemopreventive; anti-inflammatory; anti-oxidant; pro-carcinogenic	[73,74,75,76,77,78,79]
Silymarin and silibinin	Milk thistle	Silymarine:C_25_H_22_O_10_ 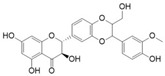 Silibinin: C_25_H_22_O_10_ 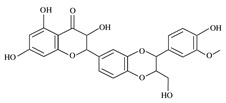	Liver diseases; chemotherapeutic; anticancer; anti angiogenic; apoptosis inducer	[80,81,82,83,84,85,86,87,88,89,90,91,92,93]
Epigallocatechin-3-Gallate	Green tea	C_22_H_18_O_11_ 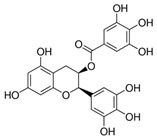	Anti-inflammatory; anti-proliferative; anticancer; anti-oxidant; anti-erythema	[94,95,96,97,98,99,100,101]
Eugenol	Cinnamon, basil, bay leaves, cloves, nutmeg	C_10_H_12_O_2_ 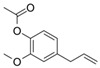	Antioxidant; anti-proliferative; Ros scavenger; anti-inflammatory; cell death inducer	[102,103,104,105]
Caffeic acid phenethyl ester	Propolis	C_17_H_16_O_4_ 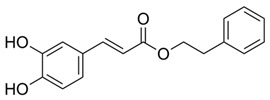	Anticancer; anti-inflammatory; immunomodulatory; anti-oxidant	[106,107,108,109]
Luteolin	Celery, olives, carrots, peppers	C_15_H_10_O_6_ 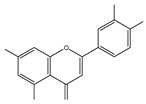	Anti-angiogenic; Apoptosis inducer; cancer cells sensitizer; stimulates melanogenesis; cell growth inhibitor	[110,111,112,113,114]
[6]-Gingerol	*Zingiber officinale*	C_17_H_26_O_4_ 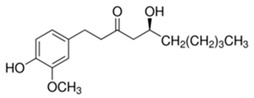	Anti-inflammatory; anti-oxidant; Ros scavenger	[115,116,117,118,119]
Caffeic acid	Coffee, vegetables, fruits	C_9_H_8_O_4_ 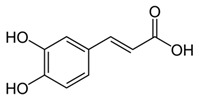	Antioxidant; anti-inflammatory; anticancer; anti-migratory; inhibits EMT	[120,121,122,123,124,125,126,127,128]

**Table 2:** Experimental approaches to analyze the effects of fruits and vegetables on skin cancer.

	In Vitro Approaches			
**Constituent**	**Major Constituents and Their Chemical and Structural Formula**	**Dose**	**Experimental Approach and Results**	**Reference**
Apple peel extract	Quercetin-3-O-β-d-glucopyranoside (C_21_H_20_O_12_) 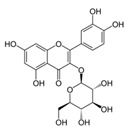	1:10–1:640	JB6/AP/kB (50% inhibition in the number of papillomas, accompanied by a decrease in tumor volume)	[189,191]
Pomegranate extract	Delphinidin-3,5-diglucoside (C_29_H_35_ClO_17_) 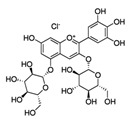	10–40 µg/ml	HaCaT cell lines (photoprotective activity against UVB-induced skin damage)	[192,193]
Pomegranate extract, oil, or juice	Delphinidin-3,5-diglucoside (C_29_H_35_ClO_17_) 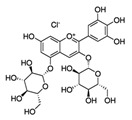	5–10 μg (extract), 0.1 mL/well (oil), 1–2 μL (juice)	Reconstituted human skin (epidermTM FT-200) (protective effect on UVB-mediated damage)	[193,194]
Grape seed extract	Catechin (C_15_H_14_O_6_) 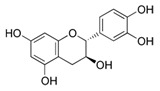	10–40 µg/ml	HaCaT cell lines (photochemopreventive action against UVB-induced skin cancer)	[195,196,197]
	**In Vivo Approaches**			
**Constituent**	**Major Constituents and Their Chemical and Structural Formula**	**Dose**	**Experimental Approach and Results**	**Reference**
Apple peel extract	Quercetin-3-O-β-d-glucopyranoside (C_21_H_20_O_12_) 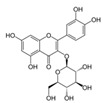	oral, in lieu of drinking water and topical application, 6 × daily	AP-1 luciferase reporter transgenic mice (C57BL/6 × DBA2) (50% inhibition in the number of papillomas, accompanied by a decrease in tumor volume)	[189,191]
		oral, in lieu of drinking water, 48 h prior	AP-1 luciferase reporter transgenic mice (C57BL/6 × DBA2) (50% inhibition in the number of papillomas, accompanied by a decrease in tumor volume)	
Pomegranate fruit extract	Delphinidin-3,5-diglucoside (C_29_H_35_ClO_17_) 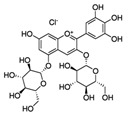	2 mg (topical), 30 min prior	CD-1 (anti-tumor; anti proliferative; anti-migratory effects)	[193,198,199,200,201]
		2 mg (topical), prior to TPA		
		0.2% in drinking water, 14 days prior	SKH-1 hairless mice (anti-tumor; anti proliferative; anti-migratory effects)	
		0.2% in drinking water, 14 days prior up to the end of treatment		
Pomegranate seed oil	Delphinidin-3,5-diglucoside (C_29_H_35_ClO_17_) 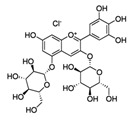	5% (topical), 1 h prior	CD-1 (anti-tumor; anti proliferative; anti-migratory)	[193,199]
Tangerine tomato powder	Vitamin C (C_6_H_8_O_6_) 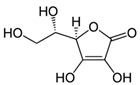	10% per os in AIN-93GA diet, 10 weeks prior	SKH-1 hairless mice (photoprotective; anti-inflammatory; anti-tumor)	[202,203,204]
Tangerine or red tomato powder	Vitamin C (C_6_H_8_O_6_) 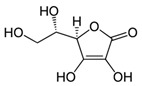	10% per os in AIN-93GA diet, through 35 weeks		[203,204,205]
Grape stem extract	Gallic acid (C_7_H_6_O_5_) 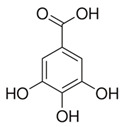	1 mg in 0.2 mL propylene glycol (Topical), daily	BALB/c mice (protection against chronic UV-induced skin damage)	[206,207,208]
Grape seed proanthocyandins	Catechin (C_15_H_14_O_6_) 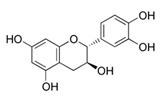	0.2–0.5% per os in AIN-76A diet	SKH-1 hairless mice (protection against UV induced skin cancer)	[196,197,209]
Grape powder	Catechin (C_15_H_14_O_6_) 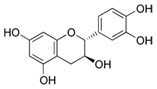	1–4 mg (Topical), 30 min post	SENCAR (inhibition of skin carcinogenesis)	[196,197,210]

## Abbreviations

4EBP	Eukaryotic initiation factor 4E (eIF4E)–binding proteins
5LOX	5-lipoxygenase
8-oxodG	8-Oxo-2′-deoxyguanosine
A375	Human melanoma cell line
A431	Human epidermoid carcinoma cell line
AIN-93G	Mice diet
AK	Actinic keratosis
Akt	Protein kinase B
AP-1	Activator protein 1
AQP3	Aquaporin 3
Bax	Bcl-2-associated X protein
BCC	Basal cell carcinoma
Bcl2	B-cell lymphoma 2protein
Bcl-3	B-cell lymphoma 3-encoded protein
CAPE	Caffeic acid phenethyl ester
CD4	Cluster of differentiation 4
CDK4	Cyclin-dependent kinase 4
CDKN2	Cyclin-dependent kinase inhibitor 2A
CDKs	Cyclin-dependent kinases
COX-1	Cyclooxygenase 1
COX-2	Cyclooxygenase 2
CPD	Carboxypeptidase D
CRP	C-Reactive Protein
CycD1	Cyclin D1
DMBA	7,12-Dimethylbenz[a]anthracene
E2F1	Transcription factor E2F1
E-cadherin	Epithelial cadherin
EGCG	Epigallocatechin gallate
EGFR	Epidermal growth factor receptor
ERK	Extracellular signal-regulated kinase
GIT	Gastrointestinal tract
GP	Grape powder
GPx	Glutathione peroxidase
GST	Glutathione S-transferase
HA14-1	Ethyl-2-amino-6-bromo-4-(1-cyano-2-ethoxy-2-oxoethyl)-4H-chromene-3-carboxylate
HaCaT	Aneuploid immortal keratinocyte cell line
H-ras	Transforming protein p21
I3C	Indole-3-carbinol
IL-1β	Interleukin-1 beta
IL-6	Interleukin-6
ILGF-1	Insulin-like growth factor 1
iNOS	Inducible nitric oxide synthase
IRS-1	Insulin receptor substrate 1
J/m^2^	Joule per square metre
JAK-2	Janus Kinase 2
JNK	c-Jun *N*-terminal kinase
LRP	Lipoprotein receptor-related proteins
MAPK	Mitogen-activated protein kinase
MDA	3,4-Methylene dioxy amphetamine
MDR-1	Multidrug resistance protein 1
MEK	Mitogen-activated protein kinase kinase
miRNA	microRNA
MITF	Microphthalmia-associated transcription factor
MMP-9	Matrix metallopeptidase 9
MPO	Myeloperoxidase
mPTP	Mitochondrial permeability transition pore
mTOR	The mammalian target of rapamycin
*N*-cadherin	Neural cadherin
NER	Nucleotide excision repair
NF-κB	Nuclear factor kappa B
NHEK	Normal human epidermal keratinocytes
NMSCs	Nonmelanoma skin cancers
ODC	Ornithine decarboxylase
p21	Cyclin-dependent kinase inhibitor 1 or CDK-interacting protein 1
p38	p38 mitogen-activated protein kinase
p53	TP53 or tumor protein
p65	nuclear factor NF-kappa-B p65 subunit
PARP	Poly (ADP-ribose) polymerase
PCNA	Proliferating cell nuclear antigen
PFE	Pomegranate fruit extract
PGE_2_	Prostaglandin E_2_
PI3-K	Phosphoinositide 3-kinase
PKB	Protein kinase B, also known as Akt
ROS	Reactive oxygen species
RV	Resvertrol
SCC	Squamous cell carcinoma
SOD	Superoxide dismutase
STAT3	Signal transducer and activator of transcription 3
TGF-β	Transforming growth factor beta 1
TPA	12-O-tetradecanoylphorbol-13-acetate
TRAF6	Tumor necrosis factor receptor (TNFR)-associated factor 6
TRK	Tyrosine kinases
UA	Ursolic acid
UV	Ultraviolet
UVA	Ultraviolet A
UVB	Ultraviolet B
VEGF	Vascular endothelial growth factor
WNT	Wingless-related integration site
